# Editorial: Recreational team sports: prevention, treatment, and rehabilitation of non-communicable diseases

**DOI:** 10.3389/fpubh.2025.1549065

**Published:** 2025-01-16

**Authors:** Malte Nejst Larsen, Magni Mohr, Felipe Lobelo, Jennifer K. Frediani

**Affiliations:** ^1^Department of Sports Science and Clinical Biomechanics, SDU Sport and Health Sciences Cluster (SHSC), Faculty of Health Sciences, University of Southern Denmark, Odense, Denmark; ^2^Faculty of Education, University of the Faroe Islands, Tórshavn, Faroe Islands; ^3^Hubert Department of Global Health, Rollins School of Public Health, Emory University, Atlanta, GA, United States; ^4^Department of Academic Advancement, Nell Hodgson Woodruff School of Nursing, Emory University, Atlanta, GA, United States

**Keywords:** team sports, health, physical activity, children, older adult

Participation in team sports is widely recognized as a cornerstone of physical, mental, and social wellbeing across all stages of life. From children to older adults, engaging in team sports fosters not only physical fitness but also resilience, social connections, and improved mental health. This *editorial* aims to highlight the role recreational team sports has as a powerful tool for promoting lifelong health, and to summarize the findings from the present Research Topic on *Recreational Team Sports: Prevention, Treatment, and Rehabilitation of Non-Communicable Diseases*.

Non-communicable diseases (NCDs) such as cardiovascular diseases and type 2 diabetes are the leading cause of death and disability worldwide, accounting for over 70% of global mortality (1). Addressing this growing burden requires innovative, accessible, and sustainable approaches to prevention, treatment. Rehabilitation and recreational team sports present an underutilized yet highly effective strategy to combat NCDs, offering physical, mental, and social benefits that support health across all stages of disease progression.

Recreational team sports provide a fun and engaging way to promote regular physical activity, a cornerstone of NCD prevention (2). Specifically, football/soccer has been shown to improve cardiovascular, metabolic, and muscle-skeletal health at a magnitude large enough to prevent a range of NCD's.

Two papers in the present topic are related to school-based interventions (Olsen et al., Costa et al.). For children, team sports provide a vital platform for physical and social development. Regular participation enhances cardiovascular fitness, muscular strength, and bone health, laying a foundation for lifelong physical wellbeing (3) and the school as an arena for regular physical activity is important, since almost all children go to school 5 days a week. The two studies in the present topic support the hypothesis that more time allocated to physical activity can be effective for physical health and wellbeing if the teachers and pedagogues are well educated to facilitate physical activities, and if the activities have a certain intensity as shown in team sports. The study by Costa et al. shows effects on levels of physical activity (PA) and fitness, and sleep duration from a relatively small change in the school day (one extra 1 h session/week) leading to a difference of more than 2 h of sedentary behavior during the day. Students may have been inspired to be more active also outside the extra session. The study by Olsen et al. is an example of a whole school implementing extra PA at the same time and thereby coping with one of the barriers often mentioned by teachers and school leaders, that the schools don't have facilities to arrange frequent sports activities. Hopefully this can be an inspiration for many to see the possibility of implementing 3 weekly sessions at a school with one small gymnasium during winter at a Northern Atlantic Island community.

For adults, participation in team sports is a powerful means of maintaining physical fitness and mitigating the risks of chronic diseases such as cardiovascular disease, diabetes, and obesity. Engaging in regular physical activity through team sports is linked to improved metabolic health and a reduced risk of premature mortality (4). Team sports also contribute to better physical performance and endurance, which are crucial for managing the demands of daily life.

Mentally, team sports offer a valuable outlet for stress reduction and social interaction. In the context of demanding careers and busy personal lives, adults often struggle to find time for meaningful social connections. Team sports provide an opportunity to connect with others, fostering a sense of community and combating feelings of isolation (5), and provide an opportunity to reduce psychological distress through social interaction, a sense of belonging, and the mental health benefits of physical activity (6).

As people age, the importance of staying active becomes even more critical. For older adults, team sports offer an enjoyable way to maintain mobility, balance, and strength, which are vital for preventing falls and maintaining independence (7, 8). Regular physical activity through team sports is also associated with improved cognitive function and a reduced risk of neurodegenerative diseases such as dementia (9). Therefore, it is important the focus on the possibilities for older adults to play as highlighted in this topic by Lozano et al..

Socially, participation in team sports helps older adults combat loneliness and social isolation, which are significant risk factors for mental health issues and reduced life expectancy (10). The shared experiences of teamwork, coupled with the physical benefits, contribute to a higher quality of life and a greater sense of purpose in later years. In the present editorial, one paper gives an example of how football fitness can be implemented at the municipal level in older adult citizens. This paper is based on a cooperative project between the municipality, the local football club, the football association and the university (Skoradal et al.). Participation in a 12-week football fitness course improved the environmental quality of life domain and postural balance in older adults and has been an ongoing course offered for the older adults during the last 3 years, indication that this is a sustainable implementation model.

Despite the well-documented benefits of team sports, many individuals face barriers to participation, such as time constraints, financial limitations, or lack of access to facilities. Addressing these challenges requires a coordinated effort by policymakers, community organizations, and sports organizations to create inclusive opportunities for people of all ages and socioeconomic backgrounds. Subsidized programs, accessible facilities, and targeted initiatives for underserved populations can help ensure that the benefits of team sports are accessible to everyone.

## Conclusion

Team sports offer far-reaching benefits that extend beyond physical health. They foster social connections, mental wellbeing, and resilience, creating a ripple effect of positive impacts throughout the lifespan. Whether for children building a foundation for healthy development, adults managing stress and maintaining fitness, or older adults enhancing longevity and quality of life, team sports are an invaluable investment in personal and societal health. By encouraging widespread participation and breaking down barriers, we can harness the transformative power of team sports to promote a healthier, more connected world.
